# An Unbiased Two-Parameter Estimation with Prior Information in Linear Regression Model

**DOI:** 10.1155/2014/206943

**Published:** 2014-06-11

**Authors:** Jibo Wu

**Affiliations:** Department of Mathematics and KLDAIP, Chongqing University of Arts and Sciences, Chongqing 402160, China

## Abstract

We introduce an unbiased two-parameter estimator based on prior information and two-parameter estimator proposed by Özkale and Kaçıranlar, 2007. Then we discuss its properties and our results show that the new estimator is better than the two-parameter estimator, the ordinary least squares estimator, and explain the almost unbiased two-parameter estimator which is proposed by Wu and Yang, 2013. Finally, we give a simulation study to show the theoretical results.

## 1. Introduction

Consider the following linear regression model:

(1) $$\begin{array}{c} Y=X\beta +\epsilon , \end{array}$$

where *Y* shows an *n* × 1 vector of observations on the dependent variable, *X* shows an *n* × *p* known design matrix of rank *p*, *β* shows a *p* × 1 vector of unknown regression coefficients, and *ϵ* shows an *n* × 1 vector of disturbances with *E*(*ϵ*) = 0 and variance-covariance matrix Cov(*ϵ*) = *σ* ^2^ *I*_*n*_.

As we all know, the ordinary least squares (OLS) estimator ${\hat{\beta }}_{\text{OLS}}={\left(X^{\prime }X\right)}^{-1}X^{\prime }Y$ has been regarded as the best estimator for a long time. However, when the multicollinearity occurs, the OLS estimator is no longer a good estimator. To treat this problem, many approaches have been presented. One method is to consider the biased estimator, such as Hoerl and Kennard [1], Swindel [2], Farebrother [3], Liu [4], Sakallog lu and Akdeniz [5], Özkale and Kaçıranlar [6, 7], Yang and Chang [8], and Wu and Yang [9, 10]. Although these biased estimators can treat multicollinearity, these estimators have big bias. In order to reduce the bias, Crouse et al. [11] and Sakallog lu and Akdeniz [5] based on ridge estimator and Liu estimator proposed the unbiased ridge estimator and unbiased Liu estimator with prior information, respectively. The unbiased ridge estimator and unbiased Liu estimator not only can deal with multicollinearity, but also have no bias.

In this paper, we will introduce an unbiased two-parameter estimator with prior information and show some properties of the new estimator.

The reminder of this paper is organized as follows. In Section 2, we give the unbiased two-parameter estimator and comparisons with OLS, two-parameter estimator proposed by Özkale and Kaçıranlar [7], and almost unbiased two-parameter estimator proposed by Wu and Yang [9] in the sense of MMSE criterion. The estimators of the parameters *k* and *d* are proposed in Section 3. A simulation study is given to explain the theoretical results in Section 4 and some conclusion remarks are given in Section 5.

## 2. Analysis of Unbiased Two-Parameter Estimator with Prior Information

In this section, we also consider the general linear regression model (1) and thus the ${\hat{\beta }}_{\text{OLS}}\sim N(\beta ,\sigma^{2}S^{-1})$ for *S* = *X*′*X*.

Crouse et al. [11] presented the unbiased ridge estimator based on the ridge estimator and prior information *J*, which is defined as follows:

(2) $$\begin{array}{c} \hat{\beta }\left(kI,J\right)={\left(X^{\prime }X+kI\right)}^{-1}\left(X^{\prime }Y+kJ\right) \end{array}$$

with *J* being uncorrelated with ${\hat{\beta }}_{\text{OLS}}$ and *J* ~ *N*(*β*, *V*). In (2), *V* = (*σ* ^2^/*k*)*I*. And in (2) the prior information *J* is a random vector for specified mean and covariance.

The two-parameter estimator proposed by Özkale and Kaçıranlar [7] is defined as follows:

(3) $$\begin{array}{c} \hat{\beta }\left(k,d\right)={\left(X^{\prime }X+kI\right)}^{-1}\left(X^{\prime }Y+kd{\hat{\beta }}_{\text{OLS}}\right)=F_{kd}{\hat{\beta }}_{\text{OLS}}, \end{array}$$

where ${\hat{\beta }}_{\text{OLS}}$ is the OLS estimator, *F*_*kd*_ = (*X*′*X* + *kI*)^−1^(*X*′*X* + *kd* *I*), and *k* > 0, 0 < *d* < 1.

Based on the two-parameter estimator, Wu and Yang [9] proposed an almost unbiased two-parameter estimator:

(4) $$\begin{array}{c} {\hat{\beta }}_{\text{AUTP}}\left(k,d\right)=\left[I-k^{2}{\left(1-d\right)}^{2}{\left(X^{\prime }X+kI\right)}^{-2}\right]{\hat{\beta }}_{\text{OLS}}. \end{array}$$

Now we study the following convex estimator:

(5) $$\begin{array}{c} \hat{\beta }\left(C,J\right)=C{\hat{\beta }}_{\text{OLS}}+\left(I-C\right)J, \end{array}$$

with *C* presenting a *p* × *p* matrix and *I* showing the *p* × *p* identity matrix. Then we can compute the mean squared error (MSE) of $\hat{\beta }(C,J)$:

(6) $$\begin{array}{c} \text{MSE}\left\{\hat{\beta }\left(C,J\right)\right\}=\sigma^{2}CS^{-1}C^{\prime }+\left(I-C\right)V{\left(I-C\right)}^{\prime }. \end{array}$$

Now we find a matrix such that $\text{MSE}\{\hat{\beta }(C,J)\}$ reaches a minimum. Solve

(7) $$\begin{array}{c} \frac{\partial \text{MSE}\left\{\hat{\beta }\left(C,J\right)\right\}}{\partial C}=2C\left(\sigma^{2}S^{-1}+V\right)-2V=0. \end{array}$$

Then we obtain *C* = *V*(*σ* ^2^ *S* ^−1^ + *V*)^−1^. Accordingly, we get *V* = *σ* ^2^(*I* − *C*)^−1^ *CS* ^−1^.

Now we can define the following estimator:

(8) $$\begin{array}{c} \hat{\beta }\left(F_{kd},J\right)=F_{kd}{\hat{\beta }}_{\text{OLS}}+\left(I-F_{kd}\right)J=\hat{\beta }\left(k,d\right)+\left(I-F_{kd}\right)J. \end{array}$$

Hence, for optimal value of *C* under the minimum MSE, the optimal convex estimator $\hat{\beta }(C,J)$ is an unbiased estimator of *β*.

For (8), since *F*_*kd*_ = (*X*′*X* + *kI*)^−1^(*X*′*X* + *kd* *I*), then we get *V* = (*σ* ^2^/*k*(1 − *d*))(*S* + *kd* *I*)*S* ^−1^. Then *J* ~ *N*(*β*, (*σ* ^2^/*k*(1 − *d*))(*S* + *kd* *I*)*S* ^−1^) for *k* > 0, 0 < *d* < 1.

For (8), it is easy to see that $\hat{\beta }(F_{kd},J)$ is an unbiased estimator of *β* and we call this estimator as UTP estimator.

Then in the following section we will give the comparisons of the new estimator with the OLS estimator, the TP estimator, and the AUTP estimator in the matrix mean squared error. Firstly, we give the definition of the matrix mean squared error (MMSE).

The matrix mean squared error (MMSE) is denoted as follows:

(9) MMSE(b)=E{(b−β)(b−β)′}=D(b)+[bias(b),bias(b)]′,

where *b* shows an estimator of *β* and *D*(*b*) and bias(*b*) present the dispersion matrix and bias vector of *b*, respectively.

The mean squared error is denoted as MSE(*b*) = tr⁡{MMSE(*b*)}.

**Lemma 1**

Let *b*_1_ and *b*_2_ be two estimators of *β*. Then *b*_2_ is called MMSE superior to *b*_1_ if

(10) $$\begin{array}{c} MMSE\left(b_{1}\right)-MMSE\left(b_{2}\right)\geq 0. \end{array}$$

**Lemma 2 (see [12])**

Let *M* be a positive definite matrix, namely, *M* > 0, and let *α* be some vector; then *M* − *αα*′ ≥ 0 if and only if *α*′*M* ^−1^ *α* ≤ 1.

**Lemma 3 (see [13])**

Suppose that *M* is a positive definite matrix and *N* is an nonnegative definite matrix; then

(11) $$\begin{array}{c} M-N\geq 0⟺\lambda_{\max}\left(NM^{-1}\right)\leq 1. \end{array}$$

### 2.1. Comparison of the OLS Estimator and the Unbiased Two-Parameter (UTP) Estimator

Now we compare the unbiased two-parameter (UTP) estimator with the OLS estimator in the matrix mean squared error (MMSE) sense.

**Theorem**

The unbiased two-parameter estimator always dominates the OLS estimator in the MMSE sense for *k* > 0 and 0 < *d* < 1.

**Proof**

Since

(12) $$\begin{array}{c} D\left({\hat{\beta }}_{\text{OLS}}\right)=\sigma^{2}S^{-1},\;\;\text{bias}\left({\hat{\beta }}_{\text{OLS}}\right)=0,\\ D\left[\hat{\beta }\left(F_{kd},J\right)\right]=\sigma^{2}{\left(S+kI\right)}^{-1}\left(S+kdI\right)S^{-1},\\ \text{bias}\left[\left(F_{kd},J\right)\right]=0, \end{array}$$

so from the definition of MMSE, we have

(13) $$\begin{array}{c} \text{MMSE}\left({\hat{\beta }}_{\text{OLS}}\right)=\sigma^{2}S^{-1}, \end{array}$$

(14) $$\begin{array}{c} \text{MMSE}\left[\hat{\beta }\left(F_{kd},J\right)\right]=\sigma^{2}{\left(S+kI\right)}^{-1}\left(S+kdI\right)S^{-1}. \end{array}$$

Then from (13) and (14), we obtain that

(15) MMSE(β^OLS)−MMSE[β^(Fkd,J)]  =σ2S−1−σ2(S+kI)−1(S+kdI)S−1  =σ2k(1−d)S−1(S+kI)−1,

is a nonnegative definite matrix for *k* > 0 and 0 < *d* < 1.

The proof of Theorem 4 is completed.

### 2.2. Comparison of TP Estimator and the Unbiased Two-Parameter (UTP) Estimator

Now we state the following theorem to compare the unbiased two-parameter estimator (UTP) with the TP estimator in the sense of MMSE.

**Theorem**

The unbiased two-parameter estimator (UTP) is superior to the TP estimator in the sense of MMSE if and only if

(16) $$\begin{array}{c} \beta^{\prime }\left(S+kdI\right)S\beta >\frac{\sigma^{2}}{k\left(1-d\right)}. \end{array}$$

**Proof**

From the definition of the MMSE, we have

(17) MMSE[β^(k,d)] =D[β^(k,d)]+[bias(β^(k,d)),bias(β^(k,d))]′ =σ2(S+kI)−1(S+kdI)S−1(S+kdI)(S+kI)−1  +k2(1−d)2(S+kI)−1ββ′(S+kI)−1.

Thus, from (14) and (17), we obtain

(18) MMSE[β^(Fkd,J)]−MMSE[β^(k,d)]=σ2(S+kI)−1(S+kdI)S−1 −[σ2(S+kI)−1(S+kdI)S−1(S+kdI)(S+kI)−1   +k2(1−d)2(S+kI)−1ββ′(S+kI)−1]=k(1−d)(S+kI)−1[σ2(S+kdI)S−1−k(1−d)ββ′] ×(S+kI)−1.

Since *k* > 0, 0 < *d* < 1 and using Lemma 2, we obtain that

(19) $$\begin{array}{c} \text{MMSE}\left[\hat{\beta }\left(F_{kd},J\right)\right]-\text{MMSE}\left[\hat{\beta }\left(k,d\right)\right] \end{array}$$

is nonnegative definite matrix if and only if

(20) $$\begin{array}{c} \beta^{\prime }\left(S+kdI\right)S\beta \leq \frac{\sigma^{2}}{k\left(1-d\right)}. \end{array}$$

So we can conclude that the unbiased two-parameter estimator (UTP) is superior to the TP estimator in the sense of MMSE if and only if

(21) $$\begin{array}{c} \beta^{\prime }\left(S+kdI\right)S\beta >\frac{\sigma^{2}}{k\left(1-d\right)}. \end{array}$$

### 2.3. Comparison of AUTP Estimator and the Unbiased Two-Parameter (UTP) Estimator

Now we state the following theorem to compare the unbiased two-parameter estimator (UTP) with the AUTP estimator proposed by Wu and Yang [9] in the sense of MMSE.

**Theorem 6**

If *λ*_max⁡_([*I* − *k* ^2^(1 − *d*)^2^(*S* + *kI*)^−2^][*I* − *k* ^2^(1 − *d*)^2^(*S* + *kI*)^−2^](*S* + *kI*)(*S* + *kd* *I*)^−1^) ≤ 1, the unbiased two-parameter estimator (UTP) is superior to the AUTP estimator in the sense of MMSE if and only if

(22) b1′{(S+kI)−1(S+kdI)S−1−σ2[I−k2(1−d)2(S+kI)−2]×S−1[I−k2(1−d)2(S+kI)−2]}≥σ2.

**Proof**

By (4), we have

(23) D(β^AUTP(k,d))=σ2[I−k2(1−d)2(S+kI)−2]×S−1[I−k2(1−d)2(S+kI)−2],bias(β^AUTP(k,d))=−k2(1−d)2(S+kI)−2β.

Thus,

(24) MMSE(β^AUTP(k,d)) =σ2[I−k2(1−d)2(S+kI)−2]S−1  ×[I−k2(1−d)2(S+kI)−2]  +k4(1−d)4(S+kI)−2ββ′(S+kI)−2.

Now we consider the following difference:

(25) MMSE[β^(Fkd,J)]−MMSE(β^AUTP(k,d)) =σ2(S+kI)−1(S+kdI)S−1  −σ2[I−k2(1−d)2(S+kI)−2]  ×S−1[I−k2(1−d)2(S+kI)−2]  −k4(1−d)4(S+kI)−2ββ′(S+kI)−2 =σ2(S+kI)−1(S+kdI)S−1  −σ2[I−k2(1−d)2(S+kI)−2]  ×S−1[I−k2(1−d)2(S+kI)−2]−b1.

Since (*S* + *kI*)^−1^(*S* + *kd* *I*)*S* ^−1^ > 0 and [*I* − *k* ^2^(1 − *d*)^2^(*S* + *kI*)^−2^]*S* ^−1^[*I* − *k* ^2^(1 − *d*)^2^(*S* + *kI*)^−2^] > 0, thus by Lemma 3, if *λ*_max⁡_([*I* − *k* ^2^(1 − *d*)^2^(*S* + *kI*)^−2^][*I* − *k* ^2^(1 − *d*)^2^(*S* + *kI*)^−2^](*S* + *kI*)(*S* + *kd* *I*)^−1^) ≤ 1, then *σ* ^2^(*S* + *kI*)^−1^(*S* + *kd* *I*)*S* ^−1^ − *σ* ^2^[*I* − *k* ^2^(1 − *d*)^2^(*S* + *kI*)^−2^]*S* ^−1^[*I* − *k* ^2^(1 − *d*)^2^(*S* + *kI*)^−2^] > 0. Then by Lemma 2, if *b*_1_′{(*S* + *kI*)^−1^(*S* + *kd* *I*)*S* ^−1^ − *σ* ^2^[*I* − *k* ^2^(1 − *d*)^2^(*S* + *kI*)^−2^]*S* ^−1^[*I* − *k* ^2^(1 − *d*)^2^(*S* + *kI*)^−2^]} ≥ *σ* ^2^, the UTP is better than the AUTP estimator.

## 3. Estimation of the Parameter *k* and Parameter *d*

In this section, we discuss how to estimate the biasing parameters *k* and *d*.

### 3.1. The Estimating of the Biasing Parameter *d*

In the definition of the new estimator, the OLS ${\hat{\beta }}_{\text{OLS}}$ is independent of *J*. Then ${\hat{\beta }}_{\text{OLS}}-J\sim N(0,\sigma^{2}S^{-1}(S+kI)/k(1-d))$ and

(26) $$\begin{array}{c} E\left[\left({\hat{\beta }}_{\text{OLS}}-J\right){\left({\hat{\beta }}_{\text{OLS}}-J\right)}^{\prime }\right]=\frac{\sigma^{2}}{k\left(1-d\right)}\left[p+k\mathrm{tr}\left(S^{-1}\right)\right]. \end{array}$$

From (26), if *σ* ^2^ is known, for a fixed *k*, we can get an unbiased estimator of *d* found as follows:

(27) $$\begin{array}{c} \hat{d}=1-\frac{\sigma^{2}\left[p+k\mathrm{tr}\left(S^{-1}\right)\right]}{k\left({\hat{\beta }}_{\text{OLS}}-J\right){\left({\hat{\beta }}_{\text{OLS}}-J\right)}^{\prime }}. \end{array}$$

When *σ* ^2^ is unknown, we use the following *s* ^2^ to estimate *σ* ^2^:

(28) $$\begin{array}{c} s^{2}=\frac{{\left(Y-X{\hat{\beta }}_{\text{OLS}}\right)}^{\prime }\left(Y-X{\hat{\beta }}_{\text{OLS}}\right)}{n-p}, \end{array}$$

and then an estimate of *d* is

(29) $$\begin{array}{c} \hat{d}=1-\frac{s^{2}\left[p+k\mathrm{tr}\left(S^{-1}\right)\right]}{k\left({\hat{\beta }}_{\text{OLS}}-J\right){\left({\hat{\beta }}_{\text{OLS}}-J\right)}^{\prime }}, \end{array}$$

where tr⁡(*S* ^−1^) = ∑_*i*=1_^*p*^1/*λ*_*i*_ and *λ*_*i*_ is the eigenvalue of *S*.

Note that in (27) and (29) the estimator of *d* may be negative. So when being in this situation, one might try to denote $\hat{d}=1$. Summing up these results, the $\hat{d}$ may be presented as follows.

**Case I**

Assuming *σ* ^2^ is known,

(i) if $k({\hat{\beta }}_{\text{OLS}}-J){\left({\hat{\beta }}_{\text{OLS}}-J\right)}^{\prime }-\sigma^{2}[p+k\mathrm{tr}(S^{-1})]>0$, then
  (30) $$\begin{array}{c} {\hat{d}}^{*}=1-\frac{\sigma^{2}\left[p+k\mathrm{tr}\left(S^{-1}\right)\right]}{k\left({\hat{\beta }}_{\text{OLS}}-J\right){\left({\hat{\beta }}_{\text{OLS}}-J\right)}^{\prime }}; \end{array}$$
(ii) otherwise
  (31) $$\begin{array}{c} {\hat{d}}^{*}=1. \end{array}$$

**Case II**

Assuming *σ* ^2^ is unknown,

(i) if $k({\hat{\beta }}_{\text{OLS}}-J){\left({\hat{\beta }}_{\text{OLS}}-J\right)}^{\prime }-s^{2}[p+k\mathrm{tr}(S^{-1})]>0$, then
  (32) $$\begin{array}{c} {\hat{d}}^{*}=1-\frac{s^{2}\left[p+k\mathrm{tr}\left(S^{-1}\right)\right]}{k\left({\hat{\beta }}_{\text{OLS}}-J\right){\left({\hat{\beta }}_{\text{OLS}}-J\right)}^{\prime }}; \end{array}$$
(ii) otherwise
  (33) $$\begin{array}{c} {\hat{d}}^{*}=1, \end{array}$$

where $s^{2}={\left(Y-X{\hat{\beta }}_{\text{OLS}}\right)}^{\prime }(Y-X{\hat{\beta }}_{\text{OLS}})/\left(n-p\right)$ is an unbiased estimator of *σ* ^2^.

### 3.2. The Estimating of the Biasing Parameter *k*

From (26), if *σ* ^2^ is known, for a fixed *d*, an unbiased estimate of *k* is defined as follows:

(34) $$\begin{array}{c} \hat{k}=\frac{p\sigma^{2}}{\left(1-d\right)\left({\hat{\beta }}_{\text{OLS}}-J\right){\left({\hat{\beta }}_{\text{OLS}}-J\right)}^{\prime }-\sigma^{2}\mathrm{tr}\left(S^{-1}\right)}. \end{array}$$

When *σ* ^2^ is unknown, similarly an estimate of *k* is

(35) $$\begin{array}{c} \hat{k}=\frac{ps^{2}}{\left(1-d\right)\left({\hat{\beta }}_{\text{OLS}}-J\right){\left({\hat{\beta }}_{\text{OLS}}-J\right)}^{\prime }-s^{2}\mathrm{tr}\left(S^{-1}\right)}. \end{array}$$

Note that in (34) and (35) the estimator of *k* may be negative. So when being in this situation, one might try to denote $\hat{k}=0$. However, there always exists a *k* such that the unbiased two-parameter estimator $\hat{\beta }(F_{kd},J)$ has smaller MSE than ${\hat{\beta }}_{\text{OLS}}$. Thus, define $\hat{k}=ps^{2}/(1-d)({\hat{\beta }}_{\text{OLS}}-J){\left({\hat{\beta }}_{\text{OLS}}-J\right)}^{\prime }$. With the above discussion, $\hat{k}$ may be presented as follows.

**Case I**

Assuming *σ* ^2^ is known,

(i) if $(1-d)({\hat{\beta }}_{\text{OLS}}-J){\left({\hat{\beta }}_{\text{OLS}}-J\right)}^{\prime }-\sigma^{2}\mathrm{tr}(S^{-1})>0$, then
  (36) $$\begin{array}{c} {\hat{k}}^{*}=\frac{p\sigma^{2}}{\left(1-d\right)\left({\hat{\beta }}_{\text{OLS}}-J\right){\left({\hat{\beta }}_{\text{OLS}}-J\right)}^{\prime }-\sigma^{2}\mathrm{tr}\left(S^{-1}\right)}; \end{array}$$
(ii) otherwise
  (37) $$\begin{array}{c} {\hat{k}}^{*}=\frac{p\sigma^{2}}{\left(1-d\right)\left({\hat{\beta }}_{\text{OLS}}-J\right){\left({\hat{\beta }}_{\text{OLS}}-J\right)}^{\prime }}. \end{array}$$

**Case II**

Assuming *σ* ^2^ is unknown,

(i) if $(1-d)({\hat{\beta }}_{\text{OLS}}-J){\left({\hat{\beta }}_{\text{OLS}}-J\right)}^{\prime }-s^{2}\mathrm{tr}(S^{-1})>0$, then
  (38) $$\begin{array}{c} {\hat{k}}^{*}=\frac{ps^{2}}{\left(1-d\right)\left({\hat{\beta }}_{\text{OLS}}-J\right){\left({\hat{\beta }}_{\text{OLS}}-J\right)}^{\prime }-s^{2}\mathrm{tr}\left(S^{-1}\right)}; \end{array}$$
(ii) otherwise
  (39) $$\begin{array}{c} {\hat{k}}^{*}=\frac{ps^{2}}{\left(1-d\right)\left({\hat{\beta }}_{\text{OLS}}-J\right){\left({\hat{\beta }}_{\text{OLS}}-J\right)}^{\prime }}, \end{array}$$

where $s^{2}={\left(Y-X{\hat{\beta }}_{\text{OLS}}\right)}^{\prime }(Y-X{\hat{\beta }}_{\text{OLS}})/\left(n-p\right)$ is an unbiased estimator of *σ* ^2^. In applications there may be other estimates of *σ* ^2^ that may also be used.

It is worthwhile to point that the proposed *k* and *d* provide an unbiased two-parameter estimator of *β* while the two-parameter estimator is biased.

## 4. A Simulation Study

In this section, we will give a simulation study to explain the theoretical results. Following McDonald and Galarneau [14], the explanatory variables are produced using the following device:

(40) $$\begin{array}{c} x_{ij}=\left(1-r^{2}\right)z_{ij}+rz_{i(p+1)},\;i=1,\ldots ,n,\;\;j=1,\ldots ,p, \end{array}$$

where *z*_*ij*_ and *z*_*i*(*p*+1)_ show independent standard normal pseudorandom numbers and *r* is specified so that the correlation between any two explanatory variables is given by *r* ^2^.

And observations on the dependent variable are then produced by

(41) yi=β1xi1+β2xi2+β3xi3+β4xi4+εi, εi~N(0,σ2),yi=β1xi1+β2xi2+β3xi3+β4xi4+β5xi5+β6xi6+εi,εi~N(0,σ2).

In this paper we consider *n* = 25,50, *p* = 4,6, *σ* ^2^ = 0.1,0.25, and *r* = 0.9,0.99,0.999. The simulation study results are given in Tables 1, 2, 3, 4, 5, 6, 7, and 8. By Tables 1–8, we can conclude that (1) when multicollinearity is serve, our new estimator performs well; (2) when *σ* ^2^ is small, our new estimator performs well; (3) when *n* is small, our new estimator performs well; (4) when *p* is big, our new estimator performs well; (5) in all cases, our new estimator is better than the OLS estimator. So we can see that our new estimator not only is unbiased, but also can overcome multicollinearity. Our estimator is meaningful in practice.

## 5. Conclusion

In this paper, we introduce an unbiased two-parameter estimator with prior information. We also show the superiority of the new estimator over the OLS estimator, the TP estimator, and the AUTP estimator in the MMSE sense. Furthermore, the estimators of the biasing parameters are also discussed in this paper.

## Figures and Tables

**Table 1:** Estimated MSE values of OLS, TP, AUTP, and UTP when *n* = 25, *p* = 4, and *σ* ^2^ = 0.1.

*r* = 0.9	*k* = 0	*k* = 0.1	*k* = 0.6	*k* = 0.7	*k* = 0.8	*k* = 0.9	*k* = 0.95	*k* = 1
OLS	0.1175	0.1175	0.1175	0.1175	0.1175	0.1175	0.1175	0.1175
TP	0.1175	0.1161	0.1788	0.1996	0.2220	0.2458	0.2581	0.2707
AUTP	0.1175	0.1174	0.1185	0.1199	0.1218	0.1242	0.1256	0.1271
UTP	0.1175	0.1151	0.1067	0.1054	0.1042	0.1032	0.1026	0.1022

*r* = 0.99	*k* = 0	*k* = 0.1	*k* = 0.6	*k* = 0.7	*k* = 0.8	*k* = 0.9	*k* = 0.95	*k* = 1

OLS	1.125	1.125	1.125	1.125	1.125	1.125	1.125	1.125
TP	1.125	1.079	2.225	2.400	2.556	2.696	2.760	2.821
AUTP	1.125	1.102	1.605	1.741	1.872	1.999	2.060	2.120
UTP	1.1250	0.9789	0.8028	0.7902	0.7799	0.7714	0.7676	0.7641

*r* = 0.999	*k* = 0	*k* = 0.1	*k* = 0.6	*k* = 0.7	*k* = 0.8	*k* = 0.9	*k* = 0.95	*k* = 1

OLS	11.236	11.236	11.236	11.236	11.236	11.236	11.236	11.236
TP	11.236	7.659	8.510	8.559	8.598	8.629	8.643	8.655
AUTP	11.24	10.33	11.88	11.98	12.07	12.13	12.16	12.19
UTP	11.236	7.627	6.930	6.905	6.886	6.870	6.864	6.858

**Table 2:** Estimated MSE values of OLS, TP, AUTP, and UTP when *n* = 25, *p* = 4, and *σ* ^2^ = 0.25.

*r* = 0.9	*k* = 0	*k* = 0.1	*k* = 0.6	*k* = 0.7	*k* = 0.8	*k* = 0.9	*k* = 0.95	*k* = 1
OLS	0.2937	0.2937	0.2937	0.2937	0.2937	0.2937	0.2937	0.2937
TP	0.2937	0.2854	0.3243	0.3417	0.3611	0.3820	0.3930	0.4043
AUTP	0.2937	0.2934	0.2913	0.2919	0.2929	0.2945	0.2955	0.2966
UTP	0.2937	0.2879	0.2667	0.2635	0.2606	0.2579	0.2566	0.2554

*r* = 0.99	*k* = 0	*k* = 0.1	*k* = 0.6	*k* = 0.7	*k* = 0.8	*k* = 0.9	*k* = 0.95	*k* = 1

OLS	2.813	2.813	2.813	2.813	2.813	2.813	2.813	2.813
TP	2.813	2.360	3.089	3.237	3.371	3.492	3.549	3.602
AUTP	2.813	2.727	3.021	3.137	3.252	3.365	3.420	3.474
UTP	2.813	2.447	2.007	1.975	1.950	1.928	1.919	1.910

*r* = 0.999	*k* = 0	*k* = 0.1	*k* = 0.6	*k* = 0.7	*k* = 0.8	*k* = 0.9	*k* = 0.95	*k* = 1

OLS	28.09	28.09	28.09	28.09	28.09	28.09	28.09	28.09
TP	28.09	15.45	14.92	14.93	14.93	14.93	14.93	14.93
AUTP	28.09	23.85	24.14	24.20	24.24	24.28	24.30	24.31
UTP	28.09	19.07	17.33	17.26	17.21	17.18	17.16	17.14

**Table 3:** Estimated MSE values of OLS, TP, AUTP, and UTP when *n* = 50, *p* = 4, and *σ* ^2^ = 0.1.

*r* = 0.9	*k* = 0	*k* = 0.1	*k* = 0.6	*k* = 0.7	*k* = 0.8	*k* = 0.9	*k* = 0.95	*k* = 1
OLS	0.0347	0.0347	0.0347	0.0347	0.0347	0.0347	0.0347	0.0347
TP	0.0347	0.0351	0.0549	0.0620	0.0700	0.0789	0.0836	0.0885
AUTP	0.0347	0.0347	0.0348	0.0348	0.0349	0.0350	0.0351	0.0351
UTP	0.0347	0.0346	0.0338	0.0337	0.0335	0.0334	0.0333	0.0333

*r* = 0.99	*k* = 0	*k* = 0.1	*k* = 0.6	*k* = 0.7	*k* = 0.8	*k* = 0.9	*k* = 0.95	*k* = 1

OLS	0.324	0.324	0.324	0.324	0.324	0.324	0.324	0.324
TP	0.324	0.339	0.931	1.068	1.202	1.332	1.396	1.458
AUTP	0.324	0.323	0.406	0.447	0.494	0.547	0.574	0.603
UTP	0.324	0.310	0.272	0.267	0.263	0.259	0.257	0.256

*r* = 0.999	*k* = 0	*k* = 0.1	*k* = 0.6	*k* = 0.7	*k* = 0.8	*k* = 0.9	*k* = 0.95	*k* = 1

OLS	3.22	3.22	3.22	3.22	3.22	3.22	3.22	3.22
TP	3.22	3.19	5.02	5.16	5.27	5.36	5.40	5.43
AUTP	3.22	3.22	5.11	5.32	5.50	5.65	5.72	5.78
UTP	3.22	2.54	2.10	2.08	2.06	2.05	2.04	2.04

**Table 4:** Estimated MSE values of OLS, TP, AUTP, and UTP when *n* = 50, *p* = 4, and *σ* ^2^ = 0.25.

*r* = 0.9	*k* = 0	*k* = 0.1	*k* = 0.6	*k* = 0.7	*k* = 0.8	*k* = 0.9	*k* = 0.95	*k* = 1
OLS	0.0868	0.0868	0.0868	0.0868	0.0868	0.0868	0.0868	0.0868
TP	0.0868	0.0867	0.1043	0.1110	0.1186	0.1270	0.1316	0.1363
AUTP	0.0868	0.0868	0.0868	0.0868	0.0868	0.0869	0.0870	0.0870
UTP	0.0868	0.0864	0.0845	0.0842	0.0838	0.0835	0.0833	0.0832

*r* = 0.99	*k* = 0	*k* = 0.1	*k* = 0.6	*k* = 0.7	*k* = 0.8	*k* = 0.9	*k* = 0.95	*k* = 1

OLS	0.809	0.809	0.809	0.809	0.809	0.809	0.809	0.809
TP	0.809	0.785	1.273	1.398	1.522	1.643	1.703	1.761
AUTP	0.809	0.807	0.866	0.902	0.945	0.993	1.019	1.046
UTP	0.809	0.776	0.679	0.667	0.656	0.647	0.643	0.639

*r* = 0.999	*k* = 0	*k* = 0.1	*k* = 0.6	*k* = 0.7	*k* = 0.8	*k* = 0.9	*k* = 0.95	*k* = 1

OLS	8.04	8.04	8.04	8.04	8.04	8.04	8.04	8.04
TP	8.04	6.20	7.08	7.18	7.26	7.32	7.35	7.37
AUTP	8.04	7.62	8.84	9.02	9.16	9.29	9.35	9.40
UTP	8.04	6.35	5.26	5.20	5.16	5.13	5.11	5.10

**Table 5:** Estimated MSE values of OLS, TP, AUTP, and UTP when *n* = 25, *p* = 6, and *σ* ^2^ = 0.1.

*r* = 0.9	*k* = 0	*k* = 0.1	*k* = 0.6	*k* = 0.7	*k* = 0.8	*k* = 0.9	*k* = 0.95	*k* = 1
OLS	0.185	0.185	0.185	0.185	0.185	0.185	0.185	0.185
TP	0.185	0.199	0.674	0.817	0.969	1.128	1.209	1.292
AUTP	0.185	0.185	0.200	0.211	0.225	0.242	0.252	0.263
UTP	0.185	0.182	0.170	0.168	0.167	0.165	0.164	0.163

*r* = 0.99	*k* = 0	*k* = 0.1	*k* = 0.6	*k* = 0.7	*k* = 0.8	*k* = 0.9	*k* = 0.95	*k* = 1

OLS	1.75	1.75	1.75	1.75	1.75	1.75	1.75	1.75
TP	1.75	2.38	8.26	9.02	9.67	10.24	10.50	10.74
AUTP	1.75	1.76	4.64	5.32	5.95	6.55	6.83	7.10
UTP	1.75	1.55	1.27	1.25	1.23	1.21	1.21	1.20

*r* = 0.999	*k* = 0	*k* = 0.1	*k* = 0.6	*k* = 0.7	*k* = 0.8	*k* = 0.9	*k* = 0.95	*k* = 1

OLS	17.4	17.4	17.4	17.4	17.4	17.4	17.4	17.4
TP	17.4	18.1	22.3	22.5	22.7	22.8	22.8	22.9
AUTP	17.4	19.7	26.8	27.1	27.4	27.7	27.8	27.9
UTP	17.4	12.0	10.8	10.7	10.7	10.7	10.7	10.7

**Table 6:** Estimated MSE values of OLS, TP, AUTP, and UTP when *n* = 25, *p* = 6, and *σ* ^2^ = 0.25.

*r* = 0.9	*k* = 0	*k* = 0.1	*k* = 0.6	*k* = 0.7	*k* = 0.8	*k* = 0.9	*k* = 0.95	*k* = 1
OLS	0.463	0.463	0.463	0.463	0.463	0.463	0.463	
TP	0.463	0.468	0.908	1.046	1.193	1.348	1.428	1.508
AUTP	0.463	0.463	0.474	0.484	0.496	0.512	0.522	0.532
UTP	0.463	0.456	0.425	0.421	0.416	0.412	0.410	0.408

*r* = 0.99	*k* = 0	*k* = 0.1	*k* = 0.6	*k* = 0.7	*k* = 0.8	*k* = 0.9	*k* = 0.95	*k* = 1

OLS	4.38	4.38	4.38	4.38	4.38	4.38	4.38	4.38
TP	4.38	4.43	9.64	10.35	10.97	11.51	11.75	11.98
AUTP	4.38	4.32	6.88	7.52	8.13	8.70	8.97	9.23
UTP	4.38	3.87	3.17	3.11	3.07	3.03	3.02	3.00

*r* = 0.999	*k* = 0	*k* = 0.1	*k* = 0.6	*k* = 0.7	*k* = 0.8	*k* = 0.9	*k* = 0.95	*k* = 1

OLS	43.6	43.6	43.6	43.6	43.6	43.6	43.6	43.6
TP	43.6	30.4	32.3	32.4	32.5	32.6	32.6	32.6
AUTP	43.6	40.9	45.8	46.1	46.4	46.5	46.6	46.7
UTP	43.6	29.9	26.9	26.8	26.7	26.7	26.7	26.6

**Table 7:** Estimated MSE values of OLS, TP, AUTP, and UTP when *n* = 50, *p* = 6, and *σ* ^2^ = 0.1.

*r* = 0.9	*k* = 0	*k* = 0.1	*k* = 0.6	*k* = 0.7	*k* = 0.8	*k* = 0.9	*k* = 0.95	*k* = 1
OLS	0.0574	0.0574	0.0574	0.0574	0.0574	0.0574	0.0574	0.0574
TP	0.0574	0.0581	0.0946	0.1076	0.1223	0.1385	0.1472	0.1563
AUTP	0.0574	0.0574	0.0575	0.0576	0.0577	0.0579	0.0580	0.0581
UTP	0.0574	0.0572	0.0559	0.0557	0.0555	0.0553	0.0551	0.0550

*r* = 0.99	*k* = 0	*k* = 0.1	*k* = 0.6	*k* = 0.7	*k* = 0.8	*k* = 0.9	*k* = 0.95	*k* = 1

OLS	0.541	0.541	0.541	0.541	0.541	0.541	0.541	0.541
TP	0.541	0.618	2.452	2.872	3.283	3.679	3.872	4.060
AUTP	0.541	0.540	0.790	0.912	1.054	1.212	1.296	1.383
UTP	0.541	0.519	0.455	0.447	0.440	0.433	0.430	0.428

*r* = 0.999	*k* = 0	*k* = 0.1	*k* = 0.6	*k* = 0.7	*k* = 0.8	*k* = 0.9	*k* = 0.95	*k* = 1

OLS	5.38	5.38	5.38	5.38	5.38	5.38	5.38	5.38
TP	5.38	7.36	14.51	15.01	15.40	15.72	15.86	15.99
AUTP	5.38	5.87	12.99	13.74	14.37	14.89	15.12	15.34
UTP	5.38	4.25	3.51	3.48	3.45	3.43	3.42	3.41

**Table 8:** Estimated MSE values of OLS, TP, AUTP, and UTP when *n* = 50, *p* = 6, and *σ* ^2^ = 0.25.

*r* = 0.9	*k* = 0	*k* = 0.1	*k* = 0.6	*k* = 0.7	*k* = 0.8	*k* = 0.9	*k* = 0.95	*k* = 1
OLS	0.144	0.144	0.144	0.144	0.144	0.144	0.144	0.144
TP	0.144	0.144	0.176	0.189	0.203	0.218	0.227	0.235
AUTP	0.144	0.144	0.144	0.144	0.144	0.144	0.144	0.144
UTP	0.144	0.143	0.140	0.139	0.139	0.138	0.138	0.138

*r* = 0.99	*k* = 0	*k* = 0.1	*k* = 0.6	*k* = 0.7	*k* = 0.8	*k* = 0.9	*k* = 0.95	*k* = 1

OLS	1.35	1.35	1.35	1.35	1.35	1.35	1.35	1.35
TP	1.35	1.37	3.03	3.43	3.82	4.20	4.39	4.57
AUTP	1.35	1.35	1.56	1.67	1.81	1.96	2.04	2.12
UTP	1.35	1.30	1.14	1.12	1.10	1.08	1.08	1.07

*r* = 0.999	*k* = 0	*k* = 0.1	*k* = 0.6	*k* = 0.7	*k* = 0.8	*k* = 0.9	*k* = 0.95	*k* = 1

OLS	13.4	13.4	13.4	13.4	13.4	13.4	13.4	13.4
TP	13.4	12.4	18.0	18.4	18.7	19.0	19.1	19.2
AUTP	13.4	13.2	19.2	19.9	20.5	21.0	21.2	21.4
UTP	13.45	10.63	8.79	8.70	8.63	8.57	8.55	8.52
